# A Global Regulatory Network for Dysregulated Gene Expression and Abnormal Metabolic Signaling in Immune Cells in the Microenvironment of Graves’ Disease and Hashimoto’s Thyroiditis

**DOI:** 10.3389/fimmu.2022.879824

**Published:** 2022-05-26

**Authors:** Haitao Zheng, Jie Xu, Yongli Chu, Wenzhou Jiang, Wenjie Yao, Shaowen Mo, Xicheng Song, Jin Zhou

**Affiliations:** ^1^ Department of Thyroid Surgery, Affiliated Yantai Yuhuangding Hospital of Qingdao University Medical College, Yantai, China; ^2^ Department of Clinical Nutrition, Affiliated Yantai Yuhuangding Hospital of Qingdao University Medical College, Yantai, China; ^3^ Department of Scientific Research, Affiliated Yantai Yuhuangding Hospital of Qingdao University Medical College, Yantai, China; ^4^ Department of Neurology, Longkou People’s Hospital, Longkou, China; ^5^ Department of Endocrinology, BinZhou Medical University, Yantai, China; ^6^ Department of Basic Science, YuanDong International Academy of Life Sciences, Nanning, China; ^7^ Department of Otorhinolaryngology, Head and Neck Surgery, Affiliated Yantai Yuhuangding Hospital of Qingdao University Medical College, Yantai, China; ^8^ Department of Endocrinology, Affiliated Yantai Yuhuangding Hospital of Qingdao University Medical College, Yantai, China

**Keywords:** Hashimoto’s thyroiditis, Graves’ disease, immune dysregulation, cell communication, thyroid tissue

## Abstract

**Background:**

Although the pathogenetic mechanisms of Hashimoto’s thyroiditis (HT) and Graves’ disease (GD) have been elucidated, the molecular mechanisms by which the abnormal immune function of cellular subpopulations trigger an autoimmune attack on thyroid tissue largely remains unexplained.

**Methods:**

The study included 2 HT patients, 2 GD patients, and 1 control donor. The thyroid samples were extracted for single-cell RNA sequencing, whole transcriptome, full-length transcriptome (Oxford Nanopore Technologies), and metabolome sequencing. Identification of immune cells with dysregulated gene expression and abnormal metabolic signaling was performed in the microenvironment, both at the bulk and single-cell levels. Based on functional enrichment analysis, the biological processes and pathways involved in abnormal immune cells were further explored. Finally, according to cell communication analysis, the global regulatory network of immune cells was constructed.

**Results:**

CD4^+^ T cells, CD8^+^ T cells, and macrophages were abnormally increased in patients with HT and GD. The differentially expressed genes of these cells were significantly involved in signaling pathways, including Th1 and Th2 cell differentiation, Th17 cell differentiation, cytokine–cytokine receptor interaction, and NF-kappa B signaling pathway. Moreover, in HT, CD4^+^ T cells interact with macrophages *via* the IL16-CCR5/FGF10-FGFR1/CXCL13-CXCR3 axis, and macrophages interact with CD8^+^ T cells *via* the CD70-CD27 axis, thereby activating the T-cell receptor signaling pathway and NF-kappa B signaling pathway. In GD, CD4^+^ T cells interact with macrophages *via* the CXCR3-CXCL10/PKM-CD44/MHCII-NFKBIE axis, and macrophages interact with CD8^+^ T cells *via* the IFNG-IFNGR1/CCR7-CCL21 axis, thereby activating T-cell receptor signaling pathway, Th1 and Th2 cell differentiation, and chemokine signaling pathway.

**Conclusion:**

In HT and GD, immune dysregulated cells interact and activate relevant immune pathways and further aggravate the immune response. This may trigger the immune cells to target the thyroid tissue and influence the development of the disease.

## Introduction

Human autoimmune thyroid disease (AITD) is the most common organ-specific autoimmune disease and the most common cause of thyroid dysfunction and nonendemic goiter, with a global incidence of more than 5% (1–3). AITD mainly manifests as Graves’ disease (GD) and Hashimoto’s thyroiditis (HT), where HT is also known as chronic lymphocytic thyroiditis (CLT) (4). Current understanding suggests that AITD is a result of a complex interaction between genetic, environmental, and endogenous factors leading to loss of self-tolerance to thyroid antigens (5). Described initially in 1912, HT is characterized by increased thyroid volume, diffuse lymphocytic infiltration, and the presence of thyroid antigen-specific antibodies (6). While GD mainly manifests as hypermetabolic syndrome caused by hyperthyroidism and is accompanied by goiter, ocular signs, and pretibial myxedema (7). Collectively, AITD has varying clinical manifestations, including euthyroidism, clinical and subclinical hypothyroidism caused by HT, clinical or subclinical hyperthyroidism of GD, and the mutual transformation of hyperthyroidism and hypothyroidism. Although HT and GD have distinct clinical features, they are identical in terms of tissue damage, including *in vivo* lymphocyte infiltration.

In HT, lymphocyte infiltration leads to thyroid cell apoptosis and hypothyroidism (8). In GD, however, similar lymphocytic infiltration results in activation of thyroid-stimulating hormone receptor (TSHR)-responsive B cells, which secrete TSHR-stimulating antibodies (TRAb). Interaction between pathogenic TRAb and TSHR, located on the thyroid follicular epithelial cell membrane, triggers an autoantigen-antibody response that leads to diffuse enlargement of thyroid tissue and excessive secretion of thyroid hormones (9). Studies have demonstrated that T lymphocytes and their specific cytokines, an indispensable part of immunity, play a critical role in the occurrence of AITD. Though infiltration of T lymphocytes leads to direct destruction of thyroid tissue, it is the presence of other T-cell subsets that synthesize proinflammatory cytokines to maintain and amplify the extent of autoimmune responses (10–13). Evolving literature elucidates the powerful role of distinct T-cell subsets in AITD, and these immunologically abnormal T-cell subsets may lead to autoimmunity to thyroid tissue (14). For example, dysregulation of regulatory T cells (Tregs) and helper T cells 17 (Th17) may stimulate the production of thyroid-stimulating antibody (TSAb), suggested as one of the factors involved in pathogenesis of GD (15, 16). In addition, Th1/Th2 imbalance also plays a key role in the pathogenesis of both HT and GD (17). While, Th1-dominated immune activity may promote apoptosis of thyroid follicular cells, leading to destruction of thyroid cells and ultimately HT, a predominant Th2-mediated immune response induces antigen-specific B lymphocyte production (TRAb), leading to GD (18, 19). However, the molecular mechanism by which the immune dysfunction of these cell subsets leads to autoimmune destruction of thyroid tissue remains largely unexplained.

In this study, based on the single-cell RNA sequencing, whole transcriptome sequencing, full-length transcriptome sequencing, and metabolome sequencing data of HT and GD, we explored the immune cells with dysregulated gene expression and abnormal metabolic signaling in the microenvironment of HT and GD, thereby constructing a global regulatory network of immune cells. This provides scientific theoretical guidance and a research basis for further understanding of the disease mechanisms mediated by immune disorders and metabolic abnormalities in HT and GD and for better intervention and treatment of the disease.

## Method

### Human Sample

The study included 2 HT patients, 2 GD patients, and 1 control donor. All the patients and the control donor agreed to a multisample library and sequencing protocol covering all study procedures. The study protocol was approved by the ethics review board of Yantai Yuhuangding Hospital. Demographic and clinical data are presented in **Supplementary Table S1**. According to the study requirement, thyroid lesions were obtained from HT and GD patients, and paranodal tissue was obtained from the control donor (who underwent surgery for suspected malignant thyroid nodules) for sequencing. RNA samples were extracted by Trizol (Thermo Fisher Scientific, Waltham, MA, USA). All sequencing was performed at Biomarker Technologies Corporation (Beijing, China).

### Single-Cell Transcriptome Sequencing

Sample preparation and cDNA library construction were performed as described in the 10× Genomics Single Cell 3′v3.1 Kit User Guide. The cDNA product and library concentrations were detected according to the qubit 4.0 fluorescence quantitative instrument, and the insertion fragment size of cDNA library was detected by qseq400 biological analyzer. Finally, the sample library was sequenced using novaseq 6000 instrument of Illumina platform. After recognizing Casava base, the obtained original image file was converted into a sequence file and stored in fastq format. The 10× genomics official software CellRanger was then used to compare and quantify the sequencing data.

### Whole Transcriptome Sequencing

For long noncoding RNA (lncRNA) sequencing, at the request of the manufacturer, the sequencing library was generated using the NEBNext^®^ Ultra™ Directional RNA Library Prep Kit for Illumina^®^ (NEB, Beverly, MA, USA), and the index code was added to the attribute sequence of each sample. The TruSeq SR Cluster Kit v3-cBot-HS (Illumina, San Diego, CA, USA) was used to cluster the index coding samples on the acBot Cluster Generation System. Following cluster generation, the library preparation was sequenced on Illumina Hiseq platform and double terminal readings were generated. The raw data obtained were stored in fastq format. The transcriptome was then assembled using StringTie (20), based on the readings mapped to the reference genome. The assembled transcripts were annotated using the gffcompare program (21).

For microRNA (miRNA) sequencing, small RNA libraries were constructed and library quality was assessed according to the manufacturer’s instructions. Subsequently, the clustering of the index-coded samples was performed on a cBot Cluster Generation System using the TruSeq PE Cluster Kit v4-cBot-HS (Illumina, San Diego, CA, USA). Following cluster generation, the library preparations were sequenced on an Illumina platform and single-end readings were generated. The raw data obtained were stored in fastq format. Considering Homo_sapiens.GRCh38_release95 as the reference genome, the unannotated readings were aligned with the reference genome using the Bowtie software (22).

### Full-Length Transcriptome Sequencing

The total RNA (1 µg) was prepared for the cDNA library using the cDNA PCR Sequencing Kit (SQK-PCS109) protocol provided by Oxford Nanopore Technologies (ONT). The cDNA library was added to FLO-MIN109 flowcells and run on the PromethION platform. The Guppy software in the MinKNOW2.2 software package was used for base calling, and the fast5 format data was converted to fastq format for storage. Subsequently, the full-length sequence was aligned with the reference genome using minimap2 software (23). Following clustering through the alignment information, the consensus sequence was obtained using pinfish software. The consensus sequences of each sample were merged and aligned with the reference genome through minimap2.

### Metabolome Sequencing

Metabolites were extracted from the samples, and supernatants were isolated for sequencing, as required for the experiment. The LC/MS system for metabolomics analysis was composed of Waters Acquity I-Class PLUS ultra-high performance liquid tandem Waters Xevo G2-XS QT of high-resolution mass spectrometer. The column used was purchased from the Waters Acquity UPLC HSS T3 column (1.8 µm, 2.1 × 100 mm).

The positive ion mode consisted of mobile phase A (0.1% formic acid aqueous solution) and mobile phase B (0.1% formic acid acetonitrile). Similarly, negative ion mode consisted of mobile phase A (0.1% formic acid aqueous solution) and mobile phase B (0.1% formic acid acetonitrile). Moreover, the injection volume was 1 μl.

Subsequently, the raw data collected with MassLynx V4.2 were processed by Progenesis QI for peak extraction, peak alignment, and other data processing operations, based on the Progenesis QI online METLIN database (24) and Biomark’s self-built library for identification, and at the same time, theoretical fragment identification and mass deviation. All were within 100 parts per million (ppm). Further analysis was performed after normalizing the original peak area information with the total peak area.

### Differential Expression Analysis

To explore the dysregulated genes of HT and GD, we performed differential expression analysis with the Limma package (25), and *p* < 0.05 and |log fold change (logFC)| >0.5 were considered significant.

### Functional Enrichment and Gene Enrichment Analysis

To further explore the biological processes (BP) and pathways involved in dysregulated genes, differentially expressed genes (DEGs) were subjected to BP analysis and Kyoto Encyclopedia of Genes and Genomes (KEGG) enrichment analysis using the clusterProfiler package in R language (26); *p* < 0.05 were considered significant.

Moreover, using the c2.cp.kegg.v6.2.symbols.gmt in the MsigDB V6.2 database (27) as the background set, the DEGs were subjected to Gene Enrichment Analysis (GSEA) (28). GSEA was performed using clusterProfiler package; *p* < 0.05 were considered significant.

### Immune Infiltration Analysis

With reference to a study by Charoentong et al. (29), immune infiltration analysis was performed using the GSEA method to evaluate the differences in immune infiltration between GD and HT patients and control donor, with special focus on cells with high degree of similarity to AITD, such as T cells, T helper cells, and macrophages.

### Construction of Single-Cell Atlas

The IntegrateData function of the Seurat package for R language (30) was used to merge single-cell data, and cell clustering analysis was performed according to the default parameters. The clustering results were uniformly reduced and visualized using PCA and T distributions and Stochastic Nearest-Neighbor Embedding (t-SNE), projected onto a 2-dimensional image, defined as a single-cell atlas. Moreover, the FindMarkers function of the Seurat package was used to identify DEGs for each cluster, and *p* < 0.05 and | logFC| >0.5 were considered significant. In addition, cell-type annotation was performed according to the known markers (**Supplementary Table S2**).

### Intercellular Communication Analysis

Ligand-receptor binding is one of the main forms of intercellular signal transduction. Therefore, we performed the cell communication analysis with the iTALK package in R (31). iTALK works by identifying highly or differentially expressed genes in cell clusters, with subsequent matching and pairing of these genes through a ligand receptor database to find important intercellular communication events.

### Reverse Transcription-Quantitative Polymerase Chain Reaction Analysis

The DEGs involved in the HT and GD regulatory networks were validated by quantitative polymerase chain reaction (qPCR). Trizol (Thermo Fisher Scientific, Waltham, MA, USA) was used to lyse tissues and extract total RNA. The cDNA was then synthesized according to the instructions of the kit (Tiangen, Beijing, China), and the qPCR reaction system was prepared by the qPCR Kit (Gene Star, Beijing, China), in which 2×q-PCR Mix 10 µl, upstream and downstream primers 0.5 µl each, replenish water to 20 µl. Finally, qPCR was performed using Applied Biosystems Stepone plus real-time quantitative PCR instrument (Thermo Fisher Scientific, Waltham, MA, USA) with the following conditions: predenaturation at 95°C for 1 min, followed by 40 cycles of 95°C for 15 s and 60°C for 15 s. The primers used in this study are shown in **Supplementary Table S3**.

### Data Analysis and Statistics

In this study, all analyses were performed based on the Bioinforcloud platform (http://www.bioinforcloud.org.cn).

## Result

### Patterns of Gene Expression Dysregulation in HT and GD at the Bulk Level

**Figure 1:**
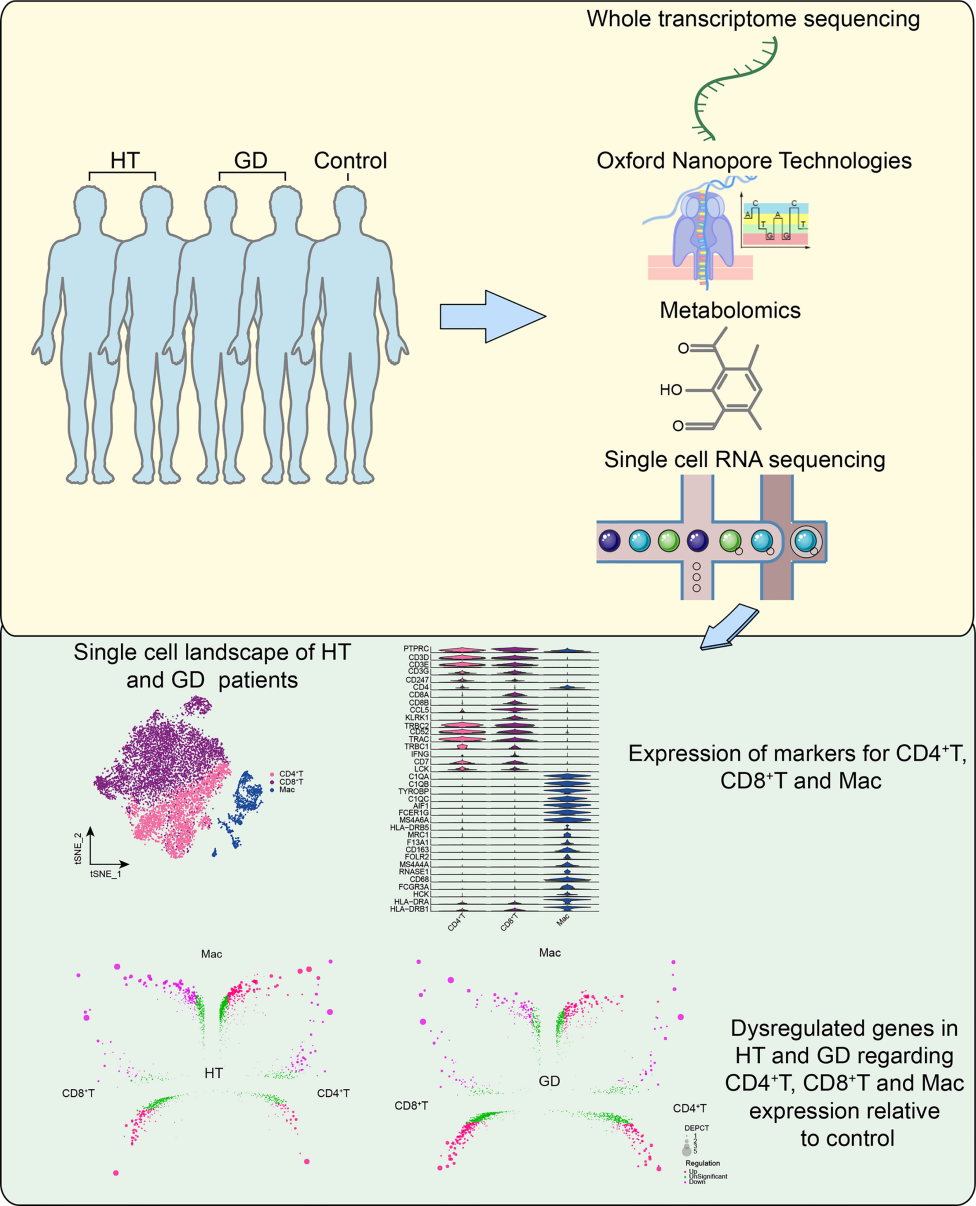
Flowchart. GD, Graves’ disease; HT, Hashimoto’s thyroiditis; Mac, macrophages.

**Figure 2:**
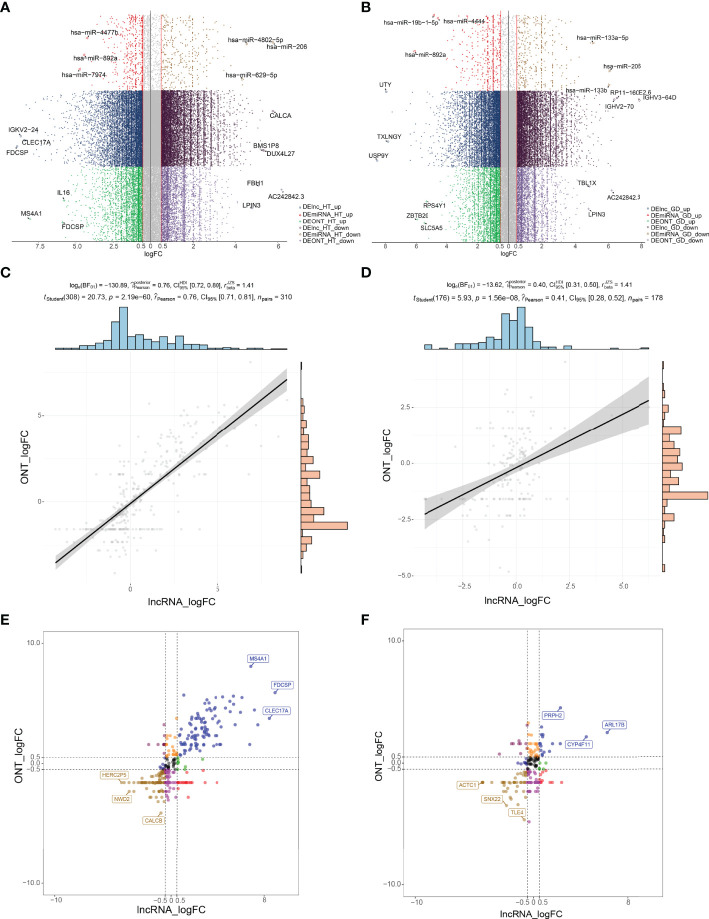
Patterns of gene expression dysregulation in HT and GD at the bulk level. **(A, B)** Manhattan plot showing DElncRNAs and DEmiRNAs in ONT sequencing and RNA sequencing in HT **(A)** and GD **(B)** patients with logFC >0.5. **(C, D)** Correlation scatter plot demonstrating the correlation of logFC of DElncRNAs and DEmRNAs in HT **(C)** and GD **(D)** patients relative to controls in ONT sequencing and RNA sequencing, *p* < 0.05. **(E, F)** Expression dysregulated genes in HT **(E)** and GD **(F)** patients; the horizontal coordinate represents the logFC of lncRNAs in RNA sequencing, the vertical coordinate represents the logFC of lncRNAs in ONT sequencing, and the genes with logFC of both >0.5 or less than −0.5 at the same time were selected to represent the high confidence expression dysregulated genes. DE, differentially expressed; GD, Graves’ disease; HT, Hashimoto’s thyroiditis; logFC, log-fold change; ONT, Oxford Nanopore Technologies.

The study flowchart is illustrated in **Figure 1**. To identify aberrantly expressed lncRNAs and miRNAs in HT and GD patients, differential expression analysis of ONT sequencing and RNA sequencing data was performed (**Supplementary Table S4**). On analysis, 6,153 lncRNAs and 741 miRNAs were found to be differentially expressed among HT patients (**Figure 2A**). Similarly, 5,492 lncRNAs and 165 miRNAs were observed to be differentially expressed among GD patients (**Figure 2B**). Moreover, the findings of correlation analysis validated the robustness of both sequencing methods used in this study (**Figures 2C, D**). Finally, we could extract genes with high confidence of dysregulated expression among HT and GD patients for follow-up analysis (**Figures 2E, F**).

### Dysregulated Gene Expression in HT and GD Is Associated With Abnormal Immune Cascades and Metabolic Signaling

**Figure 3:**
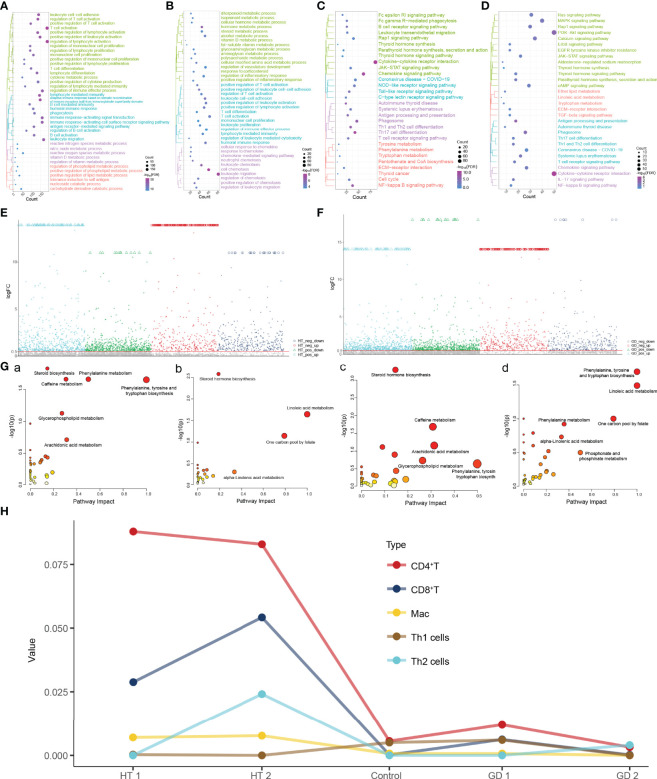
Dysregulated gene expression in HT and GD is associated with abnormal immune cascade and metabolic signaling. **(A, B)** Biological processes significantly involved in high-confidence dysregulated expression genes in HT **(A)** and GD **(B)** patients. **(C, D)** KEGG pathways significantly involved in high-confidence dysregulated expression genes in HT **(C)** and GD **(D)** patients. **(E, F)** Manhattan plot showing differential metabolites in HT **(E)** and GD **(F)** patients relative to controls, logFC >0.5. **(G)** (a) Metabolic signaling pathways activated in HT; (b) metabolic signaling pathways inhibited in HT; (c) metabolic signaling pathways activated in GD; and (d) metabolic signaling pathways inhibited in GD. **(H)** Estimation of immune infiltration in patients with HT and GD. FDR, false discovery rate; GD, Graves’ disease; HT, Hashimoto’s thyroiditis; Mac, macrophages.

To identify the molecular dysregulation mechanisms associated with HT and GD, we extracted high-confidence dysregulated genes expressed in HT and GD for enrichment analysis (**Figure 1**). HT was found to have significant leukocyte–cell adhesion, T-cell activation and differentiation, cytokine metabolic processes, and immune response-activated signal transduction of BP (**Figure 3A**). While, in GD, leukocyte–cell adhesion, hormone metabolic processes, and cell chemotaxis of BP were found to be significantly enriched (**Figure 3B**). Moreover, the KEGG signaling pathway associated with thyroid disease was found to be enriched in both HT (**Figure 3C**) and GD (**Figure 3D**). These include parathyroid hormone synthesis, secretion of thyroid hormone, and action of thyroid hormone signaling pathways and autoimmune thyroid diseases. Simultaneously, several other immune-inflammatory and metabolic signaling-related pathways were enriched in both HT and GD, including cytokine–cytokine receptor interactions, chemokine signaling pathways, Th1 and Th2 cell differentiation, NF-kappa B signaling pathways, phenylalanine metabolism, tryptophan metabolism, and tyrosine metabolism.

To further determine the role of metabolites in HT and GD, we identified differential metabolites in HT (**Figure 3E**) and GD (**Figure 3F**) relative to controls. The differential metabolites were subjected to metabolic pathway enrichment analysis, which revealed that the metabolic pathways of phenylalanine, tyrosine and tryptophan biosynthesis, phenylalanine metabolism, and steroid biosynthesis were significantly activated in HT [**Figure 3G(a)**], while the metabolic pathways of linoleic acid metabolism and steroid hormone biosynthesis were inhibited [**Figure 3G(b)**]. Moreover, steroid hormone biosynthesis, caffeine metabolism, and arachidonic acid metabolic pathways were activated in GD [**Figure 3G(c)**], while phenylalanine, tyrosine and tryptophan biosynthesis, and linoleic acid metabolic pathways were inhibited [**Figure 3G(d)**].

Given the important role of immune responses in HT and GD, we further evaluated the abundance of immune cells in the samples (**Figure 3H**), with varying degrees of infiltration of CD4^+^ T, CD8^+^ T, Mac, Th1, and Th2 cells.

### Dysregulated Gene Expression and Abnormal Metabolic Signaling in Microenvironmental CD4^+^ T Cells in HT and GD

**Figure 4:**
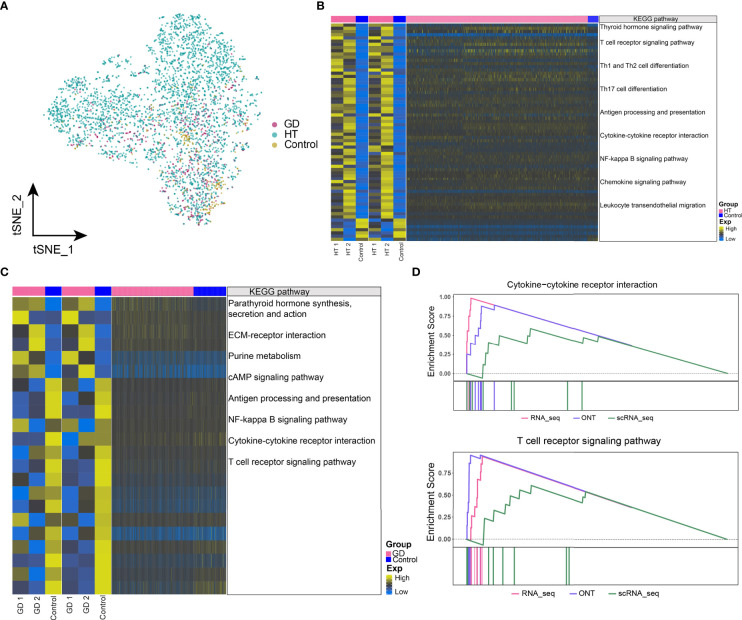
Dysregulated gene expression and abnormal metabolic signaling in microenvironmental CD4^+^ T cells in HT and GD. **(A)** Single-cell tSNE plots demonstrating HT, GD, and control microenvironment CD4^+^ T-cell profiles. **(B, C)** KEGG pathway involved in genes with high-confidence dysregulated expression in bulk level and CD4^+^ T cells in HT **(B)** and GD **(C)** patients. Heatmap of DEGs from ONT sequencing on the left, heatmap of DEGs from RNA sequencing in the middle, and heatmap of DEGs from CD4^+^ T cells on the right. **(D)** Composite GSEA plot demonstrating the KEGG pathway of significant dysregulation of microenvironmental CD4^+^ T cells in HT. EXP, expression; GD, Graves’ disease; HT, Hashimoto’s thyroiditis; KEGG, Kyoto Encyclopedia of Genes and Genomes; ONT, Oxford Nanopore Technologies.

To further understand the effect of dysregulated gene on disease at the cellular level, the abundance of CD4^+^ T cells in HT, GD, and control samples was explored, which are illustrated in **Figure 4A**. Moreover, we identified the genes that were co-overexpressed with high confidence dysregulation at the bulk level and in CD4^+^ T cells and explored the biological signatures involved in these co-disordered genes. In HT, these co-disordered genes were significantly involved in the thyroid hormone signaling pathway, T-cell receptor signaling pathway, Th1 and Th2 cell differentiation, Th17 cell differentiation, cytokine–cytokine receptor interactions, and NF-kappa B signaling pathway (**Figure 4B**). While, in GD, parathyroid hormone synthesis, secretion and action, ECM–receptor interactions, purine metabolism, cAMP signaling pathway, NF-kappa B signaling pathway, cytokine–cytokine receptor interactions, and T-cell receptor signaling pathway were significantly enriched (**Figure 4C**). In HT, GSEA demonstrated significant enrichment of cytokine–cytokine receptor interactions and T-cell receptor signaling pathways (**Figure 4D**), while no enrichment was observed in GD. Moreover, some dysregulated metabolic signaling pathways including amino acid biosynthesis and purine metabolism were found to be significantly enriched in both HT (**Supplementary Figure S1A**) and GD (**Supplementary Figure S1B**).

### Dysregulated Gene Expression and Abnormal Metabolic Signaling in Microenvironmental CD8^+^ T Cells in HT and GD

**Figure 5:**
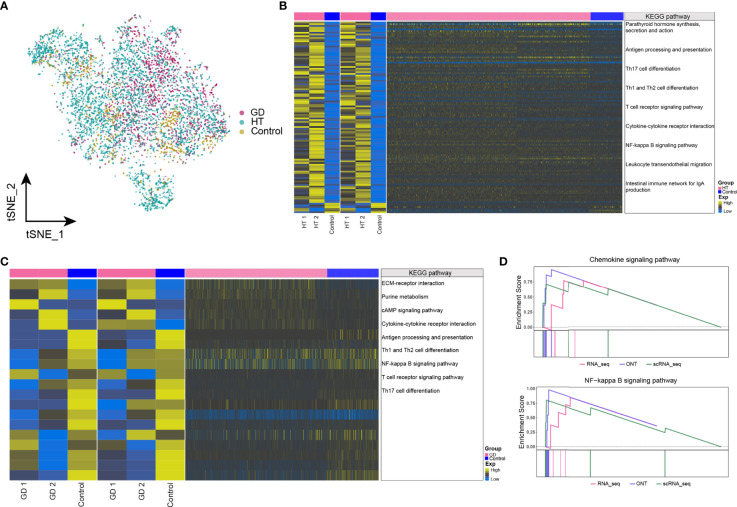
Dysregulated gene expression and abnormal metabolic signaling in microenvironmental CD8^+^ T cells in HT and GD. **(A)** Single-cell tSNE plots demonstrating HT, GD, and control microenvironment CD8^+^ T-cell profiles. **(B, C)** KEGG pathway involved in genes with high confidence of dysregulated expression at the bulk level and in CD8^+^ T cells in HT **(B)** and GD **(C)** patients. Heatmap of DEGs from ONT sequencing on the left, heatmap of DEGs from RNA sequencing in the middle, and heatmap of DEGs from CD8^+^ T cells on the right. **(D)** Composite GSEA plot demonstrating the significantly dysregulated KEGG pathway of microenvironmental CD8^+^ T cells in HT. EXP, expression; GD, Graves’ disease; HT, Hashimoto’s thyroiditis; KEGG, Kyoto Encyclopedia of Genes and Genomes; ONT, Oxford Nanopore Technologies.

The abundance of CD8^+^ T cells in HT, GD, and control is illustrated in **Figure 5A**. We identified genes that were co-overexpressed with high confidence dysregulation, both at the bulk level and in CD8^+^ T cells, and evaluated the biological signals involved in these co-disordered genes. In both HT (**Figure 5B**) and GD (**Figure 5C**), these co-disordered genes were substantially involved in antigen processing and presentation, Th1 and Th2 cell differentiation, Th17 cell differentiation, cytokine–cytokine receptor interactions, and NF-kappa B signaling pathway. In particular, synthesis, secretion, and action of parathyroid hormone as well as leukocyte migration pathways across the endothelium were enriched in HT. In GD, purine metabolism and cAMP signaling pathways were substantially enriched. In HT, GSEA revealed significant enrichment of cytokine–cytokine receptor interactions and NF-kappa B signaling pathways (**Figure 5D**), while similar enrichment was not observed in GD. However, purine metabolic signaling pathways were enriched in both HT (**Figure S2A**) and GD (**Figure S2B**).

### Dysregulated Gene Expression and Abnormal Metabolic Signaling in Microenvironmental Mac Cells in HT and GD

**Figure 6:**
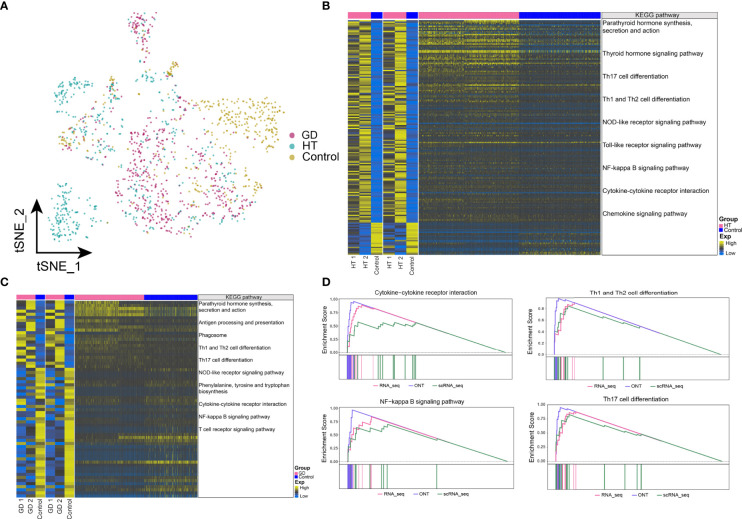
Dysregulated gene expression and abnormal metabolic signaling in microenvironmental macrophages in HT and GD. **(A)** Single-cell tSNE plots demonstrating HT, GD, and control microenvironmental Mac cell profiles. **(B, C)** KEGG pathway involved in genes with high confidence of dysregulated expression at the bulk level and in Mac cells in HT **(B)** and GD **(C)** patient. Heatmap of DEGs from ONT sequencing on the left, heatmap of DEGs from RNA sequencing in the middle, and heatmap of DEGs from Mac cells on the right. **(D)** Composite GSEA plot demonstrating the biological signal of significant dysregulation of microenvironmental Mac cells in HT. EXP, expression; GD, Graves’ disease; HT, Hashimoto’s thyroiditis; KEGG, Kyoto Encyclopedia of Genes and Genomes; ONT, Oxford Nanopore Technologies.

In particular, we found significant differences in the abundance of Mac cells in HT, GD, and control (**Figure 6A**). By exploring the biological signatures involved in genes with high confidence of dysregulated expression at the bulk level and in Mac cells, in both HT (**Figure 6B**) and GD (**Figure 6C**), we observed significant involvement of these co-disordered genes in synthesis, secretion, and action of parathyroid hormone, antigen processing and presentation, Th1 and Th2 cell differentiation, Th17 cell differentiation, NOD-like receptor signaling pathway, the NF-kappa B signaling pathway, and cytokine–cytokine receptor interactions. While in HT, the thyroid hormone signaling pathways and Toll-like receptor signaling pathways were significantly enriched. Phagosomal, phenylalanine, tyrosine, and tryptophan biosynthetic pathways were found to be significantly enriched in GD. In HT, GSEA revealed significant enrichment of cytokine–cytokine receptor interactions, NF-kappa B signaling pathway, Th1 and Th2 cell differentiation, and Th17 cell differentiation pathways (**Figure 6D**), while similar enrichment was absent in GD. Moreover, the purine and phenylalanine metabolic signaling pathways were enriched in HT (**Supplementary Figure S3A**) and GD (**Supplementary Figure S3B**), respectively.

### Global Regulatory Network of T Cells and Macrophages in the Microenvironment of HT and GD

**Figure 7:**
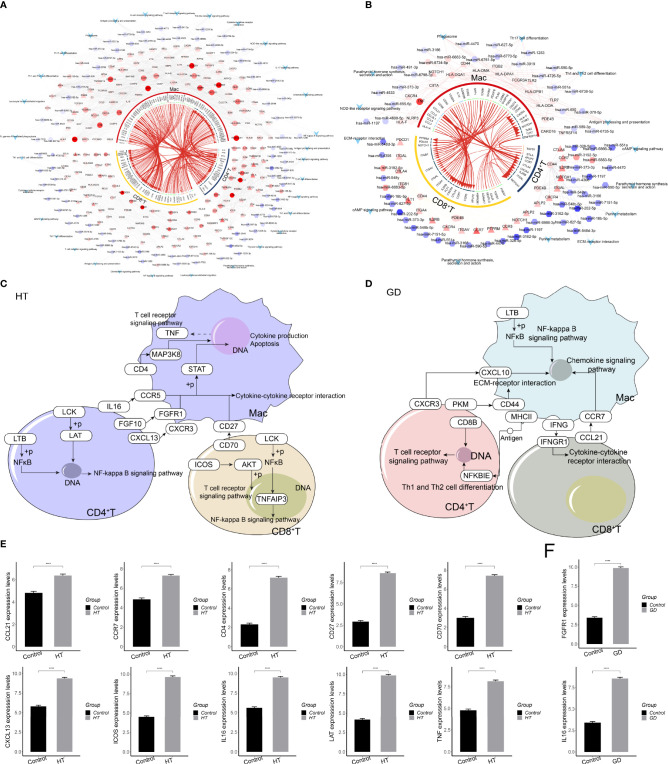
Global regulatory network of microenvironmental T cells and macrophages in HT and GD. **(A, B)** Global regulatory network of communication between microenvironmental T cells and macrophages in HT **(A)** and GD **(B)** patients. Circles represent ligand genes, triangles represent receptor genes, hexagons represent miRNAs, diamonds represent TFs, and V shapes represent signaling pathways. **(C, D)** In HT **(C)** and GD **(D)** patients, the global dysregulation mechanism of microenvironmental T cells and macrophages. **(E, F)** qPCR of genes involved in HT **(E)** and GD **(F)** regulatory networks. GD, Graves’ disease; HT, Hashimoto’s thyroiditis. ****P < 0.0001.

To determine the mechanism of intercellular regulation of the microenvironment of HT and GD, we analyzed the intercellular communication of CD4^+^ T, CD8^+^ T, and Mac cells. This led us to explore the differentially expressed ligands and receptors in different cell types, thereby allowing us to construct a comprehensive regulatory network that might be the mechanism leading to HT and GD. iTALK analysis revealed the presence of a significant difference in the abundance of ligand–receptor pairs in both HT (**Figure 7A**) and GD (**Figure 7B**). Finally, we mapped the regulatory network of dysregulated gene expression between microenvironment T cells and macrophages in HT and GD. In HT, microenvironmental T cells target macrophages *via* the IL16-CCR5 axis, FGF10-FGFR1 axis, CXCL13-CXCR3 axis, and CD70-CD27 axis, activating cytokine–cytokine receptor interactions and T-cell receptor signaling pathways (**Figure 7C**), whereas, in GD, microenvironmental T cells target macrophages *via* the CXCR3-CXCL10 axis, PKM-CD44 axis, and CCL21-CCR7 axis, activating EMC receptor interaction and NF-kappa B signaling (**Figure 7D**). Furthermore, qPCR confirmed that some of the genes involved in the HT (**Figure 7E**) and GD (**Figure 7F**) regulatory networks were differentially expressed compared to the control.

## Discussion

Previous studies have shown that abnormal immune cells may play a key role in the induction of HT and GD (17–19). In this study, based on the bulk level, we identified the dysregulated genes in HT and GD patients. Simultaneously, we observed that these genes are mainly involved in Th1 and Th2 cell differentiation, Th17 cell differentiation, and the NF-kappa B signaling pathway, as reported in previous studies. In HT patients, infiltration of the thyroid gland by Th17 cells significantly raised serum IL-17 levels (32), and the imbalance of Th1/Th17 differentiation of peripheral blood mononuclear cells may be associated with the pathogenesis of HT (33). In particular, the differentiation of Th17 lymphocytes and the synthesis of Th17 cytokines were found to be enhanced in AITD and HT, respectively (34). Moreover, we observed that HT patients had an imbalance of Th1/Treg, Th2/Treg, and Th17/Treg lymphocytes at the level of transcription factors and shifted towards Th1, Th2, and Th17 cells, respectively (35). In GD patients, the NF-kappa B signaling pathway was found to be significantly activated (36). The pathogenesis of GD may be related to the reduced Tregs and the increased IL-17 gene expression (37). This demonstrates that HT and GD patients have abnormal immune cells.

Furthermore, by combining transcriptome and metabolome, we identified immune cells with dysregulated gene expression and abnormal metabolic signaling in the microenvironment of HT and GD patients at the single-cell level. In both GD and HT, phenylalanine, tyrosine, and tryptophan biosynthesis were significantly activated, while linoleic acid metabolism and one carbon pool by folate were inhibited. We also observed that CD4^+^ T cells, CD8^+^ T cells, and macrophages were abnormally higher in HT. Similarly, CD4^+^ T cells and CD8^+^ T cells were abnormally higher in GD. Studies have reported that the cellular immune process mediated by CD4^+^ and CD8^+^ lymphocyte subsets plays an important role in the pathogenesis of HT (38). In contrast, GD revealed an increase in both CD4^+^ and CD8^+^ T cells, while HT revealed a reduced number of CD4^+^ T cells and an increase in CD8^+^ T cells (39). Moreover, cytokines produced by macrophages, T cells, and thyroid follicular cells play an important role in HT, especially in the development and perpetuation of autoimmune diseases (40). To further explore the molecular mechanisms involved in dysregulated immune cells, we explored the biological and metabolic signaling pathways involved in significantly dysregulated genes in CD4^+^ T cells, CD8^+^ T cells, and macrophages at single-cell level. Similar to the bulk level, together, these cells were significantly involved in pathways such as Th1 and Th2 cell differentiation, Th17 cell differentiation, and NF-kappa B signaling. Among them, the NF-kappa B signaling pathway has long been regarded as a typical proinflammatory signaling pathway (41), and NF-kappa B is a key signaling element of autoimmunity and an important target for the treatment of autoimmune diseases (42). Thus, activation of this pathway suggests an inflammatory response in patients, which may be a sign of immune dysregulation.

Subsequently, based on cellular communication analysis, we constructed a global regulatory network of dysregulated immune cells in HT and GD patients and observed extensive intercellular communication between CD4^+^ T cells, CD8^+^ T cells, and macrophages. Notably, these cells can interact with chemokine receptors and ligands such as CXCR3-CXCL13, CXCR3-CXCL10, and CCR7-CCL21. Among them, CXCR3 and its chemokines CXCL10, CXCL9, and CXCL11 are widely involved in the pathogenesis of autoimmune diseases such as HT, GD, thyroid eye disease (TED), type 1 diabetes, and autoimmune Addison’s disease, and may be potential targets for new drugs to treat these diseases (43, 44). CCL21, a chemokine that regulates homeostatic lymphocyte migration, is involved in lymphocyte migration to the thyroid gland (45) and is important for the circulation of CCR7-expressing cells, making it a potential target for TRAb-positive GD therapy (46). Moreover, chemokines have important effects on the polarization of macrophages. Traditionally, macrophages are categorized as (47) classically activated (M1) macrophages and alternately activated (M2) macrophages. Among them, M1 macrophages exhibit greater immune functions, while M2 macrophages exhibit greater immunosuppressive functions (48). The polarization of M1 and M2 macrophages is regulated by different chemokines (49). It is speculated that, due to the influence of specific chemokines, HT and GD promote the polarization of macrophages to M1 type, resulting in transitional immunity. It is further suggested that dysregulation of Th1 and Th2 cell differentiation has critical effects on macrophages. Stimulation of resting macrophages (M0 macrophages) with Th1 cytokines (IFNG) or TLR4 ligands (LPS) has been demonstrated to induce classic M1 polarization (50). Conversely, a Th2 cytokine (IL-4) induces alternating polarization of M2 macrophages (50). This suggests the dysfunctional nature of the immune cells in patients with HT and GD. Moreover, the interaction between cells also activates associated immune pathways and triggers transitional immunity, thereby inducing the immune cells to target thyroid tissue.

Though this study reports novel findings, it has certain limitations. First, the sample included in this study was relatively small, and the analytical results obtained require further validation in a large sample. Second, although the mechanism derived in this study is based on scientific bioinformatics analytical methods, it has not been verified by molecular and cellular experiments. Thus, we plan to further expand the sample range in future studies and use molecular and cellular experiments to verify the findings.

In conclusion, this study used the combination of the transcriptome and metabolome to identify immune cells with dysregulated gene expression and abnormal metabolic signaling in the microenvironment of HT and GD at the single-cell level. Simultaneously, we constructed a global regulatory network of these cells, contributing to immune dysregulation and metabolic abnormalities mediated by HT and GD disease mechanisms to provide a new perspective.

## Data Availability Statement

The datasets presented in this study can be found in online repositories. The name of the repository and accession number can be found below: National Genomics Data Center (https://ngdc.cncb.ac.cn/); HRA002138.

## Ethics Statement

The studies involving human participants were reviewed and approved by Yantai Yuhuangding Hospital. The patients/participants provided their written informed consent to participate in this study.

## Author Contributions

All authors contributed to the study conception and design. Material preparation, data collection and analysis were performed by HZ, JX, WJ, and WY. The first draft of the manuscript was written by SM and JZ. YC helps with bioinformatics analysis and advises on revisions to study designs. XS and JZ helped with the project administration and funding acquisition. All authors read and approved the final manuscript.

## Funding

This project was supported by the National Natural Science Foundation of China (No. 81700695), Taishan Scholars Project (No. ts20190991), and China Scholarship Council (No. 201909370036).

## Conflict of Interest

The authors declare that the research was conducted in the absence of any commercial or financial relationships that could be construed as a potential conflict of interest.

## Publisher’s Note

All claims expressed in this article are solely those of the authors and do not necessarily represent those of their affiliated organizations, or those of the publisher, the editors and the reviewers. Any product that may be evaluated in this article, or claim that may be made by its manufacturer, is not guaranteed or endorsed by the publisher.
