# Supplementary material for: A Global Regulatory Network for Dysregulated Gene Expression and Abnormal Metabolic Signaling in Immune Cells in the Microenvironment of Graves’ Disease and Hashimoto’s Thyroiditis
Source: Front Immunol. 2022 May 26;13:879824. doi: 10.3389/fimmu.2022.879824 (PMC9204353; doi:10.3389/fimmu.2022.879824)
Supplement: Supplementary file 5 [file DataSheet_1.docx]

Supplementary materials

**1 Supplementary Figures and Tables**

**1.1 Supplementary Figures**


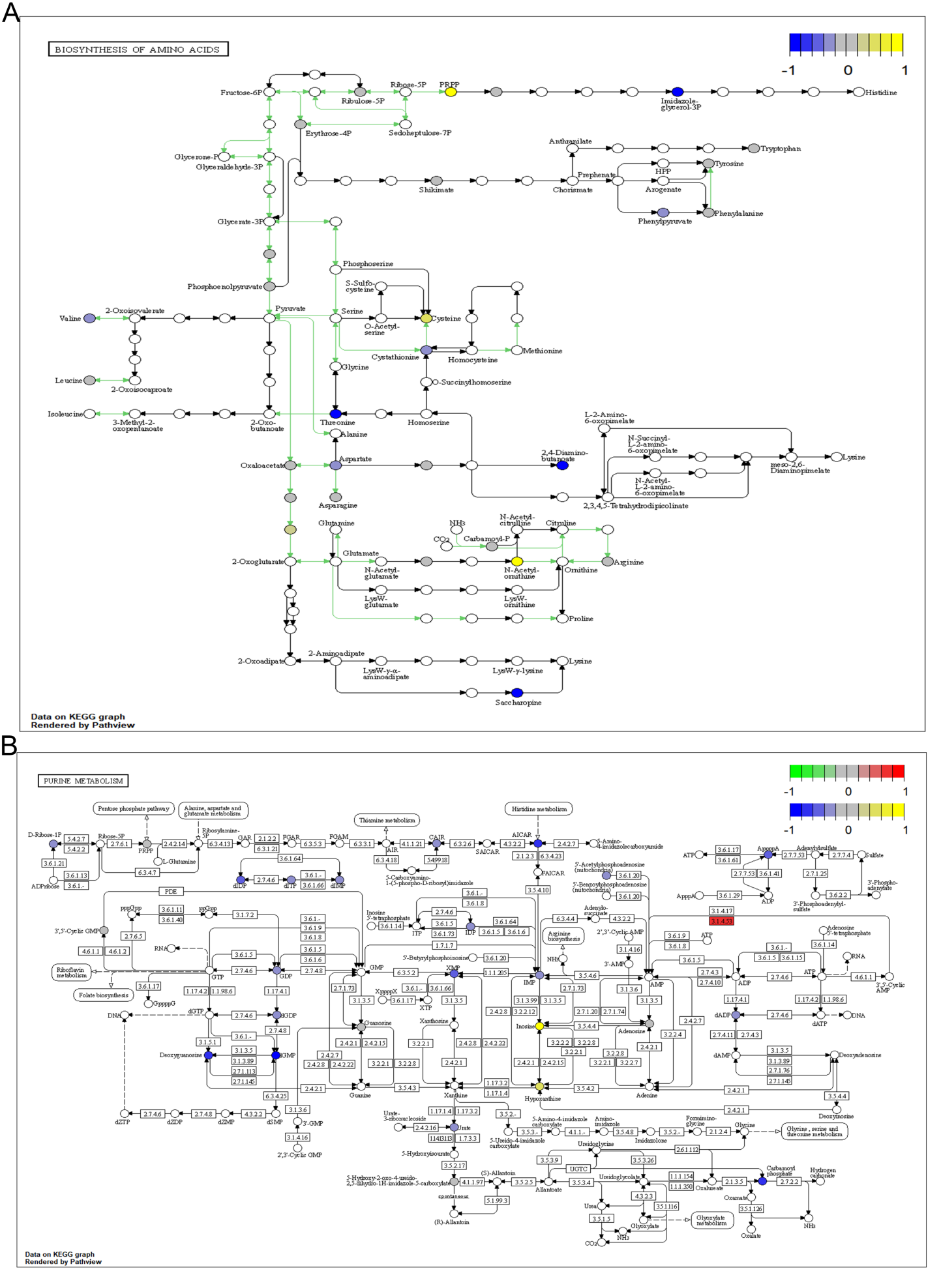


**Figure S1.** Metabolic signaling pathways significantly dysregulated in CD4^+^ T cells. A-B. Map of metabolic signaling pathways significantly dysregulated in microenvironmental CD4^+^ T cells in HT (A) and GD (B) patient.


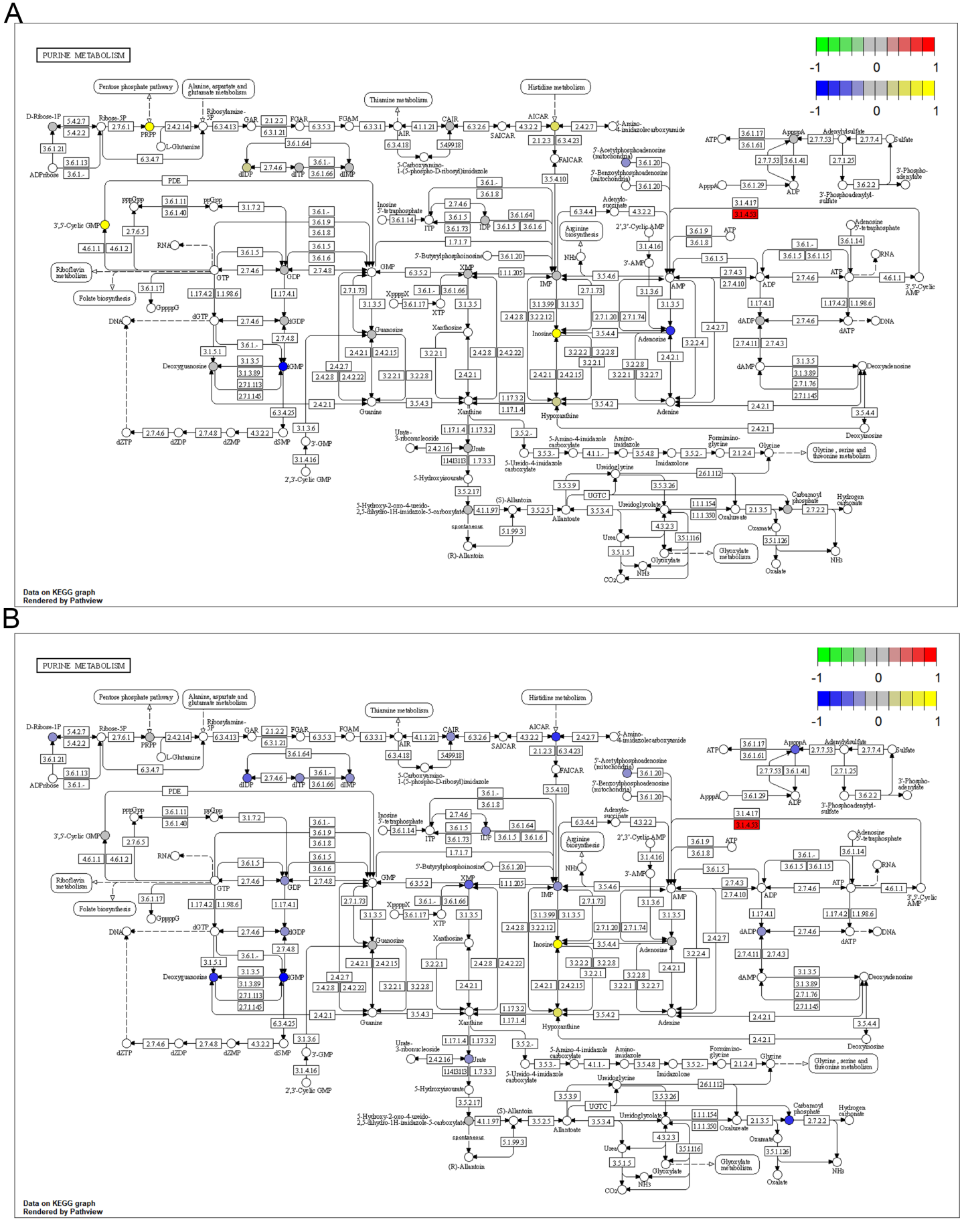


**Figure S2.** Metabolic signaling pathways significantly dysregulated in CD8^+^ T cells. A-B. Map of metabolic signaling pathways significantly dysregulated in microenvironmental CD8^+^ T cells in HT (A) and GD (B) patient.


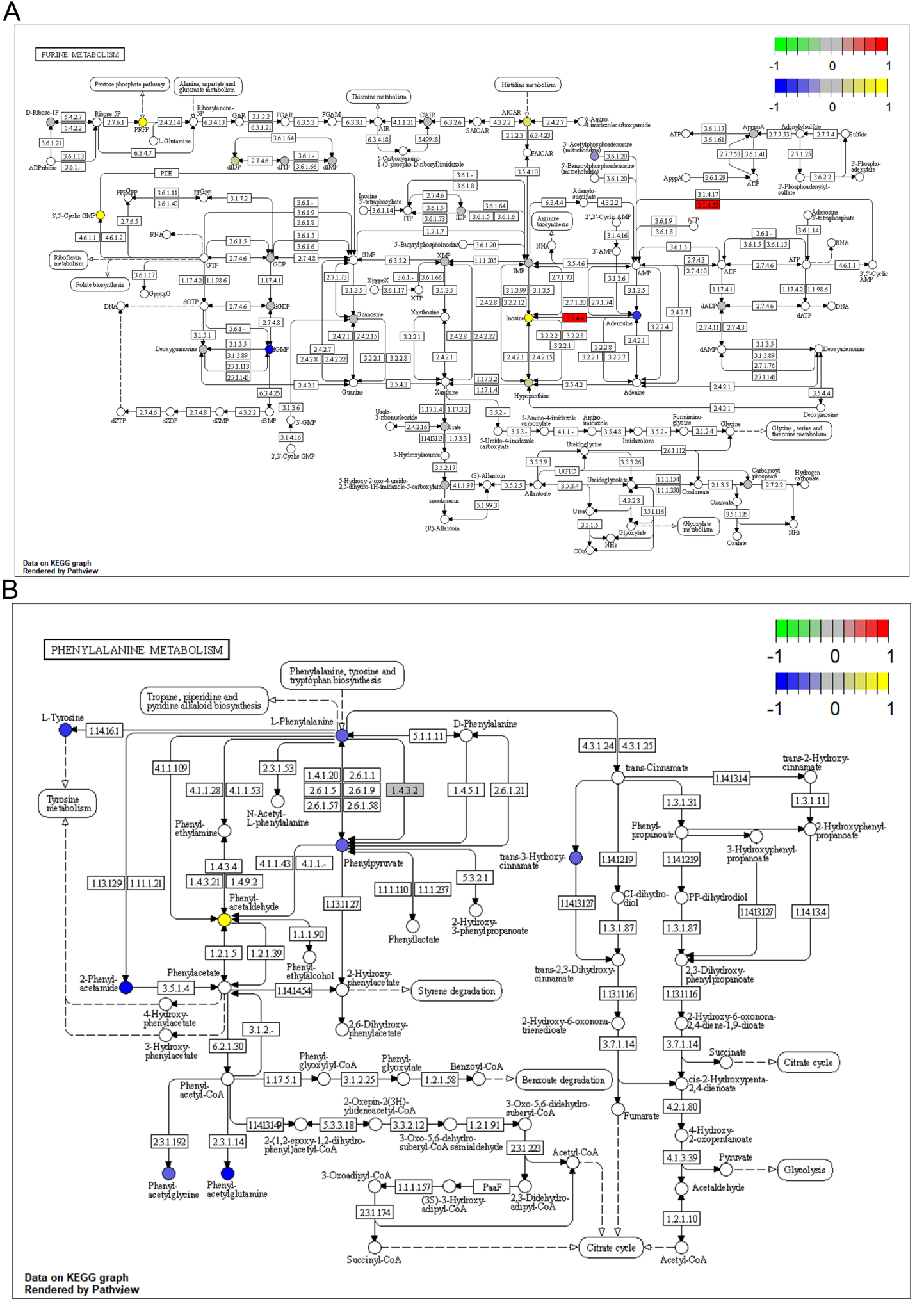


**Figure S3.** Metabolic signaling pathways significantly dysregulated in macrophages. A-B. Map of significantly dysregulated metabolic signaling pathways of microenvironmental Mac cells in HT (A) and GD (B) patient.

**1.2 Supplementary Table**

**Table S1. Demographic and clinical data.**

**Table S2. Marker genes of CD4^+^T cell, CD8^+^T cell and macrophage.**

**Table S3. Primer sequences for qPCR.**

**Table S4. Dysregulated genes in HT and GD patients.**
